# Maximizing Photosynthesis and Plant Growth in African Legumes Through Rhizobial Partnerships: The Road Behind and Ahead

**DOI:** 10.3390/microorganisms13030581

**Published:** 2025-03-04

**Authors:** Sanjay K. Jaiswal, Felix D. Dakora

**Affiliations:** Department of Chemistry, Tshwane University of Technology, Arcadia Campus, Private Bag X680, Pretoria 0001, South Africa

**Keywords:** symbiosis, water-use efficiency, food security, nodulation and N_2_ fixation

## Abstract

The interplay between soil rhizobial bacteria and leguminous plants, particularly in Africa, has a profound impact on photosynthetic efficiency and overall crop productivity. This review explores the critical role of rhizobia in enhancing photosynthesis through nitrogen fixation, a process crucial for sustainable agriculture. Rhizobial bacteria residing in root nodules provide legumes with symbiotic nitrogen that significantly boosts plant growth and photosynthetic capacity. Recent advances in molecular genomics have elucidated the genetic frameworks underlying this symbiosis, identifying key genes involved in root nodule formation and nitrogen fixation. Comparative genomics of *Bradyrhizobium* species have revealed seven distinct lineages, with diverse traits linked to nodulation, nitrogen fixation, and photosynthesis. Field studies across Africa demonstrate that rhizobial inoculation can markedly increase nodulation, nitrogen fixation, and grain yields, though outcomes vary depending on local soil conditions and legume species. Notable findings include enhanced nutrient uptake and photosynthetic rates in inoculated legumes compared with nitrate-fed plants. This review highlights the potential of utilizing indigenous rhizobia to improve photosynthesis and crop resilience. Future prospects involve leveraging genomic insights to optimize rhizobial inoculants and enhance legume productivity in water-limited environments. As climate change intensifies, integrating these advancements into agricultural practices could play a crucial role in improving food security and sustainable soil health in Africa.

## 1. Introduction

Agriculture remains the backbone of the African economy, with the majority of the population relying on farming for their livelihood [1]. However, food security in many parts of Africa is threatened by various challenges, including soil degradation, climate change, and limited access to fertilizers [2]. Legumes are a group of plants which include species like cowpea, soybean, and groundnuts that have the unique ability to fix atmospheric nitrogen biologically [3,4]. This natural process of nitrogen fixation not only enhances soil fertility, but also supports the growth of legumes in nutrient-poor soils, making them important in the context of African agriculture [5].

Biological nitrogen fixation (BNF) is a natural process by which specified microorganisms convert atmospheric nitrogen (N_2_) into ammonia (NH_3_), a form that plants can absorb and utilize for their growth. BNF is primarily carried out by two groups of microorganisms: symbiotic nitrogen fixers and free-living, non-symbiotic heterotrophic nitrogen fixers [6]. Symbiotic nitrogen-fixing bacteria, such as *Rhizobium* and *Bradyrhizobium*, form mutualistic associations with leguminous plants, as well as Frankia species that associate with non-leguminous plants like actinorhizal trees [7,8]. The bacteria inhabit specialized root structures called nodules, where nitrogen fixation takes place.

Free-living, non-symbiotic heterotrophic nitrogen fixers include microorganisms such as *Azotobacter, Clostridium*, and cyanobacteria (*Anabaena* and *Nostoc*) that fix nitrogen independently in the soil or water without forming a direct association with plants [9].

This symbiotic relationship allows legumes to thrive, even in soils with low nitrogen, thus reducing the need for synthetic nitrogen fertilizers, which are often expensive, inaccessible to smallholder farmers in Africa, and cause global warming [10].

In addition to their nitrogen-fixing ability, legume-rhizobia interactions play a critical role in enhancing photosynthesis, a process by which plants convert sunlight energy into carbohydrate [5]. Photosynthesis is thus essential for plant growth and productivity, as it provides about 90% of the carbon in plants [11]. However, it is sensitive to environmental stresses such as drought, temperature, and nutrient deficiencies, conditions that are prevalent in Africa [12]. The ability of legume–rhizobia interactions to fix nitrogen biologically can significantly influence their photosynthetic efficiency [13], as nitrogen is a key component of chlorophyll (the molecule that captures light energy during photosynthesis) and Rubisco (the enzyme that reduces CO_2_) [14,15]. Chlorophyll molecules contain four nitrogen atoms per porphyrin ring, making nitrogen an essential component for their synthesis [16]. Moreover, more than half of the N in the photosynthetic machinery of crops is allocated to the formation of Rubisco enzyme [17].

In African farming systems, where the availability of chemical fertilizers is limited and the risk of soil nutrient depletion is high, legumes provide a sustainable source of N to improve soil health and crop productivity [18]. The introduction of nitrogen-rich legume residues into crop rotations can help to restore soil fertility, reduce dependency on external inputs, and increase the resilience of cropping systems to climate variability [19,20,21]. Furthermore, legumes contribute to dietary diversity and nutritional security in African communities by providing protein-rich food sources [22].

Recent studies have suggested that legumes may have an additional advantage in the context of climate change due to their ability to mitigate photosynthetic acclimation, a phenomenon whereby plants adjust their photosynthetic functioning in response to environmental changes, sometimes leading to reduced efficiency [23]. As global temperatures rise and rainfall patterns become more unpredictable, the demand for crops that can maintain high photosynthetic rates under stress is likely to increase. Legumes, with their traits for biological nitrogen fixation, may better sustain their photosynthetic activity under adverse conditions, offering a potential buffer against the negative impacts of climate change on crop yields in Africa [24].

Despite the clear benefits of legumes in African agriculture [25,26], there remains a need for more research into optimizing their use in different agroecological zones. Understanding biological nitrogen fixation and its relationship with photosynthesis in legumes can result in strategies to enhance crop productivity and sustainability. By leveraging the natural traits and gene expression of legumes, it is possible to develop more resilient cropping systems that can support Africa’s growing population, while preserving its natural resources.

This review explores the role of nitrogen-fixing soil microbes in enhancing photosynthesis in grain legumes grown in African soils. It discusses the mechanisms through which these microbes improve plant growth and productivity, and reviews field and laboratory studies conducted in Africa, including practical applications, molecular/genomic studies, and future research directions. The findings highlight the potential of using nitrogen-fixing microbes to boost the productivity and sustainability of grain legume production in Africa.

## 2. Nitrogen-Fixing Soil Microbes: An Overview

Biological nitrogen fixation (BNF) is a natural and eco-friendly process essential for maintaining soil fertility and supporting agricultural productivity. This process is facilitated by a group of specialized diazotrophic microorganisms, which have the unique ability to convert atmospheric nitrogen (N_2_) into ammonia through the activity of nitrogenase, an enzyme complex encoded by *nif* genes [27]. Diazotrophs comprise various bacteria, including members of the Rhizobiaceae (α-proteobacteria) and Burkholderiaceae (β-proteobacteria) families, actinobacteria from the genus *Frankia*, and certain cyanobacteria from the *Nostocaceae* family [9,28,29,30]. These nitrogen-fixing microbes are able to establish symbiotic relationships with their host plants, particularly legumes, forming root nodules in which the nitrogen fixation process occurs. Among certain *Bradyrhizobium* species, the nodulation (*nod*) and nitrogen fixation (*nif*) genes exhibit significant variability [31]. For example, *Bradyrhizobium* sp. G22 lacks both the nodulation genes (*nodABC*) and the nitrogen fixation genes (*nifDKH*) that are present in *Bradyrhizobium diazoefficiens* USDA 110 [32]. Comparative genomic analyses suggest that *nod* and *nif* genes are species- and host-specific [33,34]. Previous studies have shown that these (*nod* and *nif*) genes are typically organized into clusters that regulate nodulation and nitrogen fixation [35]. Interestingly, research has revealed that some nonsymbiotic *Bradyrhizobium* isolates, which lack the *nod* gene cluster and cannot nodulate legumes, are nevertheless commonly found in soils [36,37].

The legume-rhizobia symbiosis is the most significant BNF process in agriculture, as it can supply up to 80% of the nitrogen required by leguminous crops, thereby reducing reliance on synthetic nitrogen fertilizers [38]. This symbiosis is therefore crucial for sustaining agricultural productivity and promoting soil health, especially in the nutrient-poor soils commonly found in many regions of Africa.

## 3. Photosynthesis: A Fundamental Process

Photosynthesis is the most important biological process, followed by nitrogen fixation, as it enables plants, algae, and cyanobacteria to convert light energy from the sun into chemical energy stored in organic compounds such as carbohydrates. This process not only fuels metabolism in these organisms but also plays a crucial role in maintaining the Earth’s oxygen levels by releasing oxygen as a byproduct [39].

The process of photosynthesis occurs in two stages, the light-dependent reactions and the light-independent reactions (Calvin cycle). During the light-dependent reactions, chlorophyll and other pigments within the chloroplasts absorb light energy, which is used to generate ATP and NADPH, the energy carriers. These reactions occur in the thylakoid membranes of the chloroplasts and are essential for driving the subsequent stages of photosynthesis.

In the Calvin cycle, ATP and NADPH produced during the light-dependent reactions are used to reduce atmospheric CO_2_ into organic molecules. The enzyme Rubisco plays a central role in this process by catalyzing the conversion of CO_2_ into three-carbon sugar intermediates, which are ultimately synthesized into glucose and other carbohydrates. These carbohydrates serve as energy sources for the plant and are vital for its growth and development.

**Figure 1:**
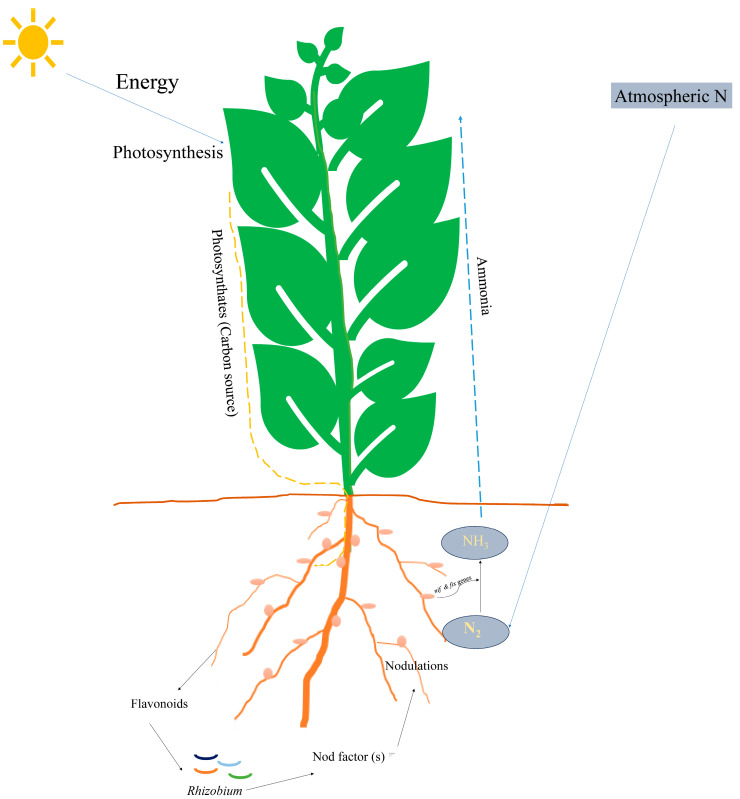
This diagram provides a schematic representation of how leguminous plants engage in a mutualistic relationship with *Rhizobium* to fix atmospheric nitrogen. The energy derived from photosynthesis drives the synthesis of photosynthates, which fuel root and nodule development and support the biological nitrogen fixation (BNF) process. Through this intricate biological system, the plant obtains essential nitrogen in a usable form ammonia, thereby enhancing its growth and productivity without reliance on external nitrogen fertilizers.

However, photosynthesis is also important for mitigating climate change as it captures CO_2_ from the atmosphere and converts it into organic matter, thus reducing atmospheric CO_2_ concentration, which is the root cause of global warming [40]. This process underpins the energy flow in ecosystems and supports the existence of virtually all life forms on Earth (Figure 1).

## 4. Role of Rhizobia in Enhancing Photosynthesis

Nitrogen is a vital nutrient for plant growth and development, and its availability significantly influences the efficiency of photosynthesis. Chlorophyll is an important nitrogenous macromolecule needed for harvesting light energy for photosynthetic functioning in algae and land plants [41]. The enzyme Rubisco (Rubilose bisphosphate carboxylase:oxygenase) is also a nitrogenous macromolecule needed to reduce CO_2_ into carbohydrates during photosynthesis. Photosynthetic functioning is therefore dependent on the adequate supply of nitrogen for the biosynthesis of chlorophyll, Rubisco, and other major enzymes involved in the Calvin cycle and plant carbohydrate metabolism. In nodulated legumes, nitrogen supply for plant growth via photosynthesis is generally from nitrogen fixation in root nodules. In fact, in Africa, many legumes can derive as much as 50–98% of their nitrogen nutrition from symbiotic fixation [42]. There is therefore a direct relationship between nitrogen fixation in legumes and photosynthesis; the higher the nitrogen fixation in legumes, the greater the supply of nitrogen for increased formation of chlorophyll and Rubisco, leading to higher photosynthetic activity. This increase in the supply of *de novo* photosynthate by the legume to nitrogen-fixing rhizobia in root nodules also further stimulates nitrogen fixation in the host plant, leading to a positive feedback loop between photosynthesis in legumes and nitrogen fixation in nodules.

Through their symbiotic relationship with legumes, soil bacteria called rhizobia play a critical role in enhancing nitrogen (N) supply to the host plant by reducing atmospheric N_2_ into ammonia, which plants can readily utilize. Additionally, nitrogen accumulation in soils occurs when symbiotically fixed N is released by decomposing plant residues, root exudates, and/or decaying nodules, thus contributing to the build-up of soil nitrogen pools [43].

Symbiotic N_2_ fixation is therefore essential for synthesizing chlorophyll, the nitrogenous pigmented macromolecule responsible for capturing light energy during photosynthesis [44]. By providing biological N, rhizobia directly contribute to the formation of chlorophyll, Rubisco, and other photosynthetic enzymes, thereby enhancing the plant’s photosynthetic capacity. Improved N supply also supports better root development for nutrient uptake and water-use efficiency, needed for overall plant health and productivity.

**Figure 2:**
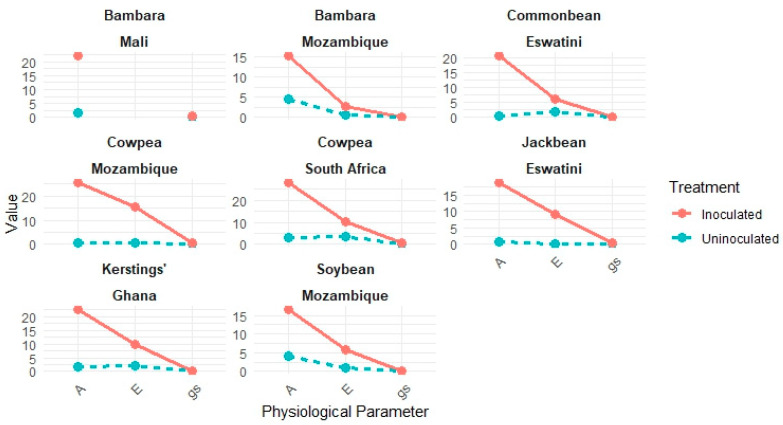
This line graph presents a comparative analysis of physiological parameters across different crops and countries under two treatments: Inoculated and Uninoculated. The parameters considered in this analysis are Photosynthesis Rate (A) (μmol CO_2_ m^−2^ s^−1^), Stomatal Conductance (gs) (mol H_2_O m^−2^ s^−1^), and Transpiration Rate (E) (mmol H_2_O m^−2^ s^−1^). The trends in this graph shows that the inoculated treatment often shows higher values for the physiological parameters compared to the uninoculated treatment, indicating the possible benefits of inoculation on crop performance. Uninoculated Treatment shows lower values, suggesting a reduced physiological response in the absence of inoculation. Graph was constructed based on published data from Ibny et al. (2019) [13]; Gyogluu et al. (2018) [46]; Dlamini et al. (2021) [47]; Mbah et al. (2022) [5]; Simbine et al. (2021) [48]; Ngwenya et al. (2022) [8]; Gunununu et al. (2023) [49].

In general, grain legumes inoculated with effective rhizobial strains exhibit higher photosynthetic rates, increased chlorophyll levels, and improved plant growth. For example, studies of Bambara groundnut, Kersting’s groundnut, and cowpea in Ghana, South Africa, and Eswatini have shown that, by enhancing N_2_ fixation, rhizobia boost photosynthetic functioning, leading to higher grain yields [5,13,45] (Figure 2).

The role of rhizobia in enhancing photosynthesis highlights the importance of this symbiotic relationship in sustainable agriculture. By reducing the need for chemical fertilizers and improving plant productivity, rhizobia contribute to more resilient and environmentally friendly cropping systems.

## 5. Nutrient Availability and Photosynthetic Efficiency in Legume–Rhizobia Symbiosis

Nutrient availability, in particular nitrogen, is crucial for optimizing photosynthetic efficiency and overall plant growth. Grain legumes like cowpea form symbiotic relationships with nitrogen-fixing rhizobia, which not only enhance nitrogen supply to the host plant, but also its nutritional profile, including increases in the concentrations of both macro- and micronutrients in edible parts, such as the leaves and grain [22]. For example, inoculating cowpea genotypes with *Bradyrhizobium* strain CB756 increased tissue concentrations of essential micronutrients, such as Fe, Zn, Mn, and Cu, in leaves and grain [5], as well as the macronutrients phosphorus (P), magnesium (Mg), sulfur (S), and calcium (Ca) in leaves, which surpassed the concentrations obtained with nitrate feeding [5]. This capability can be harnessed to produce nutrient-rich cowpea leaves and grain under controlled conditions for enhancing human nutrition and health. In addition to symbiotic N supply, rhizobia are also capable of solubilizing bound P for use by plants, producing siderophores for increased Fe availability to plants, and secreting morphogenic molecules (e.g., lumichrome, riboflavin, and indole acetic acid) for stimulating root growth and the host plant immune response [10].

Recent studies by Xiao et al. [50] have shown that, relative to non-nitrogen-fixing legumes, leaf photosynthetic capacity and stomatal conductance are influenced more by phosphorus levels in nitrogen-fixing legumes. Rhizobial strains such as TUTVUSA28, TUTVUSA41, and TUTVUSA46 have also been reported to induce higher levels of boron (B) in leaves and zinc (Zn) in the grain of legume species. Interestingly, while rhizobial inoculation generally increases micronutrient concentrations in edible organs, nitrate-fed plants sometimes exhibit higher macronutrient levels, such as P and K, suggesting different nutrient uptake and accumulation mechanisms in symbiotic versus nitrate-fed plants. High nitrogen-fixing cowpea symbioses, in particular, can accumulate significant amounts of important dietary elements in their edible leaves, making them a valuable crop for enhancing human nutrition and health [51,52].

Apart from all elements, iron (Fe) plays a major role in nitrogen fixation and photosynthesis [53,54]. Recently, Ito et al. [54] investigated the transcriptome response to nitrogen status in *Lotus japonicus*, and identified IRON MAN (IMA) peptide genes expressed during nitrogen fixation. LjIMA1 and LjIMA2, which were expressed in shoots and roots, apparently regulate systemic and local Fe accumulation in root nodules. IMA peptides also maintain the N–Fe balance in *L. japonicus*, while IMA-mediated Fe provision is reported to be essential for nitrogen-related physiological processes in legume root nodule symbiosis.

## 6. Photosynthetic Functioning and Water-Use Efficiency (WUE)

Water availability is a critical factor for crop production, particularly in smallholder farms where water scarcity can limit biomass production. Cowpea genotypes exhibit significant variation in leaf photosynthetic rates, gas exchange parameters, and intrinsic water-use efficiency (WUEi) [5,55]. Genotypes with higher photosynthetic rates tend to produce more shoot biomass, underscoring the role of photosynthesis in driving biomass accumulation. For instance, cowpea genotypes such as IT07K-299-69 and IT10K-817-3, which displayed higher photosynthetic rates, also exhibited increased stomatal conductance and leaf transpiration, leading to greater biomass production [5]. The positive correlations between photosynthesis (A), stomatal conductance (gs), and transpiration (E) highlight the importance of stomatal regulation in optimizing photosynthesis.

Recent studies have identified cowpea genotypes such as Soronko, Asetenapa, Nhyira, Omandaw, and IT90K-277-2, which exhibited high water-use efficiency in different locations in Ghana [55]. These genotypes coupled their greater WUE with higher grain yield, making them ideal candidates for breeding programs aimed at enhancing plant–water relations and grain yield [56,57]. The robustness of carbon isotope discrimination (δ^13^C) in estimating plant water-use efficiency has been established for field legumes across African countries, thus supporting its use in selecting crops with improved WUE and high grain yield under water-limited conditions [56,58].

Water-use efficiency, a measure of how effectively plants use water for carbon fixation, varied among cowpea genotypes, with genotype IT07K-299-69 exhibiting greater shoot δ^13^C, or high WUE, despite recording lower intrinsic WUE. Genotypes IT10K-866-1 and Songotra, however. showed greater shoot δ^13^C and higher WUEi, indicating the importance of WUE in maximizing biomass accumulation and grain yield, particularly in water-scarce environments.

## 7. Mechanisms of Legume–*Rhizobia* Interaction

The legume–rhizobia symbiosis is one of the most important plant–microbe interactions in sustainable agriculture, as it provides biological nitrogen to cropping systems. This symbiotic relationship is mediated by the mutual recognition of signaling molecules produced by both legumes and rhizobia. In legumes, these signals are typically flavonoids and anthocyanins released by the plant [10]. They interact with NodD proteins of compatible rhizobia to form a NodD–flavonoid complex. This complex acts as a transcriptional activator, inducing the expression of nodulation (*nod*) genes. The activation of these genes results in the synthesis of lipo-chito-oligosaccharide Nod factors, which initiate root hair curling and deformation [59].

Nitrogen-fixing soil microbes, particularly rhizobial bacteria, play a crucial role in enhancing photosynthesis in legumes through atmospheric N_2_ fixation. These bacteria convert atmospheric nitrogen (N_2_) into ammonia (NH_3_), a form that plants can readily incorporate into organic molecules, thereby boosting plant growth and photosynthetic efficiency. In return, the host plant provides *de novo* photosynthate to the N_2_-reducing bacteroids for increased N_2_ fixation [40].

Advances in genomics have shed light on the numerous genes involved in root nodule formation and N_2_ fixation, revealing how leguminous plants and their microsymbionts interact at the molecular level to facilitate N_2_ fixation [60]. This process is essential for plant health and productivity, as nitrogen is a key nutrient for chlorophyll synthesis, Rubisco enzyme formation, and overall plant growth.

Aerobic diazotrophs, including N_2_-fixing bacteria associated with legumes, utilize various mechanisms to generate low-potential electrons required for nitrogenase activity [61]. Nitrogenase, the enzyme responsible for converting N_2_ to NH_3_, requires electrons with reduction potentials of around −400 to −500 mV. In general, diazotrophs employ different strategies to produce these electrons, including substrate-level ferredoxin (Fd) oxidoreductases, hydrogenases, photosystem I or other light-driven reaction centers, electron bifurcating *fix* complexes, proton motive force-driven *Rnf* complexes, and Fd:NAD(P)H oxidoreductases [61].

These intricate mechanisms ensure that legumes receive a steady supply of ammonia, essential for synthesizing vital organic compounds, such as chlorophyll and Rubisco macromolecules. Thus, the availability of nitrogen from symbiosis directly enhances photosynthetic efficiency by providing the necessary components for chlorophyll formation, the biosynthesis of Rubisco and enzymes involved in C metabolism, as well as other critical biological processes, ultimately contributing to improved plant growth and agricultural productivity. Understanding these mechanisms is vital for enhancing N_2_ fixation efficiency in legume crops, leading to increased yields, improved soil health, and reduced reliance on chemical N fertilizers, thus supporting sustainable agricultural practices.

## 8. Molecular and Genomic Studies

Legume plants, through their interaction with rhizobial bacteria in soils, form a unique organ called the root nodule. This organ houses the rhizobial microsymbionts, which perform biological N_2_ fixation by reducing atmospheric N_2_ to NH_3_, which is converted into organic N molecules by the host plant. Recent advances in genomics have provided new insights into the complete gene inventory of these rhizobial microsymbionts. The entire genome sequences of the microsymbionts of several legume species have been determined and meticulously annotated. By combining genomic with proteomic approaches, numerous genes involved in root nodule formation and N_2_ fixation have been identified.

In recent years, the increasing availability of *Bradyrhizobium* genomes has facilitated the delineation and characterization of new species. Comparative genomic analyses have revealed that *Bradyrhizobium* can be divided into seven lineages, three of which correspond to the so-called supergroups within the genus. The wide distribution of key lifestyle traits, such as nodulation, N_2_ fixation, and photosynthesis, suggests complex evolutionary histories for these traits [60].

A recent comparative genomic study [35] identified genes linked to symbiotic N_2_ fixation, revealing that the *Bradyrhizobium* pan-genome consists of 84,078 gene families, including 824 core genes and 42,409 accessory genes. Core genes are primarily involved in crucial cellular processes, while accessory genes serve diverse functions, including nodulation and N_2_ fixation. Three distinct genetic profiles were identified based on the presence or absence of gene clusters related to nodulation, N_2_ fixation, and secretion systems. Most *Bradyrhizobium* strains from soil and non-leguminous plants lacked major *nif*/*nod* genes and were evolutionarily more closely related [35].

The *Bradyrhizobium* genus includes diverse bacteria, some of which are capable of photosynthesis [62]. Recently, comparative genomics was used to explore the distribution and evolution of photosynthesis in *Bradyrhizobium* [63], and photosynthesis gene clusters (PGCs) were found in 25 genomes across three lineages: photosynthetic, *B. japonicum*, and *B. elkanii* supergroups. Two PGC architectures were identified, with PGC1 found primarily in the photosynthetic supergroup and some *B. japonicum* strains, while PGC2 was in *B. japonicum* and *B. elkanii* strains, and resembled clusters in *Rhodopseudomonas*. Ancestral state reconstruction showed that PGCs were acquired via horizontal gene transfer, followed by vertical inheritance and losses.

The symbiotic relationships between legumes and rhizobia for nitrogen fixation can influence photosynthesis and plant metabolism [64] (Pérez-Fernández et al., 2024). A transcriptomic study by He et al. [65] explored the regulation of nodulation and photosynthesis using a non-nodulated soybean mutant. Gene mapping revealed a recessive mutation in the *rj1* locus, likely a new allele, while grafting experiments showed that nodulation is root-determined. This enhances nitrogen metabolism and photosynthesis in non-nodulated scions grafted to nodulated roots. Transcriptome analysis identified 853 and 1874 differentially expressed genes (DFGs) in leaves and roots, respectively, with notable pathways and gene ontology (GO) terms. Key DEGs highlighted the role of *nod* genes in nitrogen metabolism, hormone regulation, and photosynthesis.

## 9. Practical Applications and Field Studies

Field and laboratory studies conducted in Africa are summarized in this section to the benefits of rhizobial inoculation on photosynthetic activity and plant growth or productivity.

### 9.1. Bambara Groundnut (Vigna subterranea L. Verdc.)

Ibny et al. [13] reported significant differences in nodulation, shoot dry matter, leaf chlorophyll concentration, and photosynthetic activities induced by rhizobial isolates on Bambara groundnut in South Africa. The isolates elicited varying levels of leaf photosynthesis, stomatal conductance, and chlorophyll concentrations, based on their symbiotic effectiveness. For example, *Bradyrhizobium* strain TUTNou60, isolated from soils in Mali, induced markedly higher photosynthetic rates (22.1 µmol (CO_2_) m^−2^ s^−1^), which were accompanied by greater stomatal conductance and leaf transpiration (Figure 2). The lowest chlorophyll concentrations were found in the 5 mM KNO_3_-fed plants and the uninoculated control, which recorded the lowest stomatal conductance and photosynthetic rates (7.8 and 1.6 µmol (CO_2_) m^−2^ s^−1^, respectively). The increased photosynthetic functioning in inoculated plants was further evidenced by the significant correlation found between photosynthetic rates and leaf stomatal conductance, as well as between photosynthesis and leaf transpiration.

### 9.2. Jack Bean (Canavalia ensiformis L.)

Recently, Ngwenya and Dakora [66] reported variable nodule numbers, nodule dry matter, shoot biomass, and photosynthetic functioning in Jack bean (*Canavalia ensiformis* L.) inoculated with 22 indigenous rhizobial isolates under glasshouse conditions. Both N_2_ fixation and photosynthetic carbon assimilation differed among the test isolates according to their symbiotic effectiveness. This was evidenced by the significant correlations found between nodule number and nodule dry matter, as well as between nodule number and shoot dry mass, which suggested that enhanced N_2_ fixation by rhizobia in root nodules increased carbon accumulation via photosynthesis, leading to greater plant growth and biomass accumulation in Jack bean. Earlier studies found similar results with Bambara groundnut and Kersting’s bean [13,67]. The native isolate TUTCEeS2 from Jack bean showed symbiotically superior performance, with a very high %RSE (relative symbiotic effectiveness), which indicated its potential for use in inoculant formulation. Several studies similarly found that highly effective native rhizobia in African soils outperformed the commercial rhizobial inoculants currently in use by farmers [47,67,68]. The high photosynthetic functioning elicited by the rhizobial isolates was notable with isolate TUTCEeS11, which was phylogenetically aligned with *B. elkanii* and induced the highest photosynthetic rates (18.81 µmol (CO_2_) m^−2^ s^−1^), greater stomatal conductance, and leaf transpiration [69]. Significant differences (*p* < 0.05) in shoot %C elicited by the test isolates were related to symbiotic effectiveness, with marked variation in shoot and root biomass due to photosynthesis. A study by Adams et al. [70] found that, for most N_2_-fixing plants (N_2_FP), carbon fixation and stomatal conductance (gs) were not related to nitrogen per leaf area (N_area_). However, woody N_2_FP exhibited higher intrinsic water-use efficiency (WUEi) with increased Narea.

In a host range study by Gyogluu et al. [46], 10 native soybean rhizobia isolated from Mozambican and South African soils were tested for their symbiotic effectiveness and ability to induce high rates of photosynthesis in cowpea (*Vigna unguiculata* L. Walp.), Bambara groundnut (*Vigna subterranea* L. Verdc.), Kersting’s groundnut (*Macrotyloma geocarpum* Harm), and soybean (*Glycine max* L. Merr). The results showed that some strains were ineffective in non-homologous host plants. Where cowpea and Kersting’s groundnut were not nodulated or ineffectively nodulated, they exhibited much lower photosynthesis and stomatal conductance. Of the effectively nodulated Bambara groundnut plants by their microsymbionts, isolates TUTRLR3B, TUTM19373A, and TUTMJM5, which phylogenetically belonged to *Bradyrhizobium elkanii* group [71], elicited much greater leaf photosynthesis and stomatal conductance. With soybean, isolates TUTM19373A and TUTRLR3B, as well as TUTMJM5 and TUTMCJ7B, induced greater photosynthesis and stomatal functioning in the leaves of genotype PAN1664R.

The carbon sink strength of the rhizobial legume symbiosis is largely determined by the rate of N_2_ fixation, which is in turn stimulated by photosynthesis [72]. As a result, the photosynthetic rates induced by rhizobial isolates in their host plants were much higher than those recorded by nitrate-fed plants. These greater photosynthetic rates in nodulated plants were attributed to an increase in carbon sink strength caused by enhanced N_2_ fixation in mature root nodules, causing a rise in photosynthetic functioning. The positive effect of rhizobial inoculation on photosynthetic rates and carboxylation efficiency was previously reported in soybean [73,74]. These higher photosynthetic rates, stomatal conductance, and leaf transpiration found in nodulated plants were consistent with those in earlier reports for Bambara groundnut, cowpea, and Kersting’s groundnut [5,13,67].

### 9.3. Cowpea (Vigna unguiculata L. Walp.)

Despite the widely reported drought tolerance of cowpea [75], most cultivated varieties are yet to be assessed for their water relations in the field. In a study by Mohammed et al. [55], cowpea genotypes Asetenapa at Nyankpala, Marfotuya, Padituya, SARVx-09-004, and Zayura at Savelugu all showed greater %N and %C in shoots at those locations, suggesting that increased carbon via photosynthesis stimulated N nutrition, leading to a positive correlation between %N and %C. The functional link between N and C nutrition via photosynthesis has been reported for field-grown cowpea [76,77]. Given the rising atmospheric CO_2_ due to climate change, and the reported stimulation of N_2_ fixation under elevated CO_2_ [40], incorporating legumes into cropping systems could increase C sequestration, and reduce greenhouse gas emissions.

In South Africa, cowpea inoculated with native *Bradyrhizobium* strain TUTSAN 92.2.1 showed higher photosynthetic rates (27.81 µmol (CO_2_) m^−2^ s^−1^) than the uninoculated control (3.0 µmol (CO_2_) m^−2^ s^−1^). These indigenous isolates also elicited higher photosynthetic rates (20.57 to 27.81 µmol (CO_2_) m^−2^ s^−1^) than the nitrate-fed plants [5]. Stomatal conductance induced by the test isolates closely mirrored transpiration rates, with values ranging from 0.09 mol m^−2^ s^−1^ in the uninoculated control to 0.62 mol m^−2^ s^−1^ in plants inoculated with isolate TUTSAN 31.2.2. Transpiration rates and stomatal conductance were lowest in the uninoculated control (3.77 mmol (H_2_O) m^−2^ s^−1^) and highest in plants inoculated with isolate TUTSAN 90.3 (12.16 mmol (H_2_O) m^−2^ s^−1^).

### 9.4. Common Bean (Phaseolus vulgaris L.)

Common bean seedlings inoculated with different rhizobial isolates also induced varying levels of leaf photosynthetic rates (A) and other gas exchange parameters based on symbiotic effectiveness [78,79]. *Rhizobium* isolates TUTPvES 24(1), TUTPvES 2(3), TUTPvES 5(1), and TUTPvES 11(3) induced markedly higher photosynthetic rates (20.71, 20.61, 20.60, and 20.47 µmol (CO_2_) m^−2^ s^−1^, respectively), while stomatal conductance was greater with bean strains TUTPvES 49(2), TUTPvES 6(1), TUTPvES 59(1), and TUTPvES 11(3) (0.45, 0.45, 0.44, and 0.42 mol (CO_2_) m^−2^ s^−1^, respectively). However, the isolates that induced lower stomatal conductance and transpiration generally recorded lower photosynthetic rates, and vice versa [49].

## 10. Conclusions and Perspectives

The research reviewed here highlights the critical role of rhizobial microsymbionts in promoting N_2_ fixation, photosynthesis, and overall plant growth in different legume species. Advances in molecular and genomic studies have significantly deepened our understanding of the complex symbiotic relationships between legumes and rhizobia, particularly in the African agricultural systems. The ability of indigenous rhizobial strains to outperform commercial inoculants in enhancing photosynthetic efficiency, N_2_ fixation, and carbon accumulation underscores their potential for improving crop yields and resilience in diverse environmental conditions.

For the future, further exploration of the genetic diversity and functionality of native rhizobial strains is essential. This can lead to the development of tailored inoculants that are optimized for specific crops and environmental conditions, particularly in regions like Africa, where soils are rich in diverse, yet underutilized, rhizobial populations. Integrating genomic and proteomic approaches will be key to identifying additional genes involved in symbiosis, N_2_ fixation, and photosynthesis, which can then be targeted for enhancing crop productivity.

Field studies that assess the long-term impacts of rhizobial inoculation on plant growth, soil health, and carbon sequestration are also needed. As climate change continues to pose challenges to global agriculture, the use of rhizobia to boost the resilience and sustainability of legume crops offers a promising pathway. Selecting and utilizing legume genotypes that show enhanced nitrogen and carbon accumulation, can contribute to mitigating climate change through increased carbon sequestration and reduced greenhouse gas emissions.

## Data Availability

No new data were created or analyzed in this study.
